# Outcome benefit of abdominal paracentesis drainage for severe acute pancreatitis patients with serum triglyceride elevation by decreasing serum lipid metabolites

**DOI:** 10.1186/s12944-016-0276-6

**Published:** 2016-06-24

**Authors:** Zhu Huang, Sun-Hong Yu, Hong-Yin Liang, Jing Zhou, Hong-Tao Yan, Tao Chen, Long Cheng, Lin Ning, Tao Wang, Zhu-Lin Luo, Kui-Ying Wang, Wei-Hui Liu, Li-Jun Tang

**Affiliations:** Postgraduate Department, Third Military Medical University, Chongqing, China; General Surgery Center of PLA, Chengdu Military General Hospital, Chengdu, Sichuan Province 610083 China

**Keywords:** Severe acute pancreatitis, Abdominal paracentesis drainage, Free fatty acid

## Abstract

**Background:**

Our previous reports demonstrated that abdominal paracentesis drainage (APD) exerts a beneficial effect on severe acute pancreatitis (SAP) patients. However, the underlying mechanisms for this effectiveness are not well understood.

**Methods:**

A retrospective cohort of 132 consecutive non-hypertriglyceridemia (HTG)-induced SAP patients with triglyceride (TG) elevation and pancreatitis-associated ascitic fluid (PAAF) was recruited from May 2010 to May 2015 and included in this study. The patients were divided into two groups: the APD group (*n* = 68) and the non-APD group (*n* = 64). The monitored parameters mainly included mortality, hospital stay, the incidence of further intervention, levels of serum lipid metabolites and inflammatory factors, parameters related to organ failure and infections, and severity scores.

**Results:**

The demographic data and severity scores were comparable between the two groups. Compared with the non-APD group, the primary outcomes (including mortality, hospital stay and the incidence of percutaneous catheter drainage) in the APD group were improved. The serum levels of lipid metabolites were significantly lower in the APD group after 2 weeks of treatment than in the non-APD group. Logistic regression analysis indicated that the decreased extent of free fatty acid (FFA)(odds ratio, 1.435; *P* = 0.015) was a predictor of clinical improvement after 2 weeks of treatment.

**Conclusion:**

Treatment with APD benefits non-HTG-induced SAP patients with serum TG elevation by decreasing serum levels of FFA.

## Background

Our previous research recommended a modified step-up approach in which a new procedure, abdominal paracentesis drainage (APD), was used during the early stage of treatment for severe acute pancreatitis (SAP) [1]. We found that patients with APD had a lower rate of mortality, less organ failure and lower inflammatory factors than the non-APD group in our retrospective clinical cohort study. Furthermore, we investigated the safety of APD using a prospectively study and confirmed that there was no increase in the rate of infections with the APD procedure [2]. These previous studies suggest that APD could be beneficial in the treatment of patients with SAP and carries no additional risk of infection [3]. However, the underlying mechanisms for this effectiveness are not well understood [4].

The possible mechanisms for the effectiveness of APD could come from two sources: 1) decreasing intra-abdominal hypertension (IAH); 2) eliminating the toxic substances, mainly including inflammatory mediators, proteases and lipid metabolites in pancreatitis-associated ascitic fluid (PAAF). Among these substances, lipid metabolites, the reaction products of fat necrosis, have been demonstrated to be involved in the development and progression of pancreatitis [5–8]. In an experimental model of SAP, high concentrations of free fatty acid (FFA) have been detected in PAAF, and the lipid metabolites extracted from the PAAF could promote the exacerbation of inflammation *in vitro* [5]. Recently, FFA in necrotic collections generated from peri-pancreatic or visceral fat necrosis have been reported to be a possible mediator of the conversion of mild acute pancreatitis (AP) to SAP because of its ability to induce necro-apoptosis and cause multi-system injury [6].

In addition, triglycerides (TG), which can be transformed into to FFA, have been reported to have a mild-to-moderate elevated level in plasma (2-10 mmol/L) in approximately half of the patients with AP [9]. Similar to FFA, studies have shown that the serum TG elevation correlates with the aggravation of non-HTG-induced SAP [10–12]. For example, we recently revealed that acute biliary pancreatitis (ABP) patients with TG elevation usually had higher risks of SAP and mortality, more organ failure and a greater likelihood of requiring further intervention compared with those with normal TG levels. These abovementioned studies indicate that lipid metabolites, especially TG and FFA, are enriched in PAAF and play a specific role in the progression of SAP [12]. Although these advances in knowledge have been made, the exact roles of lipid metabolites in the effectiveness of APD have not been determined.

In this work, we aim to investigate (i) whether APD is beneficial to non-HTG-induced SAP patients with TG elevation; (ii) whether removing the PAAF and thereby removing the lipid metabolites in the fluids could reduce the level of lipid metabolites in plasma; (iii) whether the effectiveness of APD correlates with the reduction of lipid metabolites in plasma. To this aim, we undertook this retrospective clinical cohort study to assess the clinical effect of APD in non-HTG-induced SAP patients with elevated serum TG levels and PAAF and investigate the changes in the concentration of lipid metabolites after treatment.

## Methods

### Patient selection

We collected clinical data from consecutive SAP patients who were admitted to the General Surgery Center from May 2010 to May 2015. The SAP diagnosis was based on clinical findings, biochemical parameters, and the computed tomography severity index (CTSI), according to the revised Atlanta Classification [13]. The including criteria were as follows: 1) Adults (older than 18 years) diagnosed with SAP within 48 h after onset. 2) Fluid collections in the abdominal or pelvic cavity found via imaging examinations, such as computed tomography or ultrasound. 3) TG level 72 h after onset ≥1.88 mmol/L. 4) No history of hyperlipidemia or alcohol abuse. The exclusion criteria were: 1) TG level 72 h after onset ≥11.3 mmol/L. 2) Primary (genetic) or secondary disorders of lipoprotein metabolism (e.g., diabetes, obesity, hypothyroidism, drugs and so on). 3) Patients who had undergone antihyperlipidemic therapy, such as insulin and/or heparin treatment, apheresis and oral drugs during treatment. 4) Patients with a medical history of immune deficiency, previous abdominal surgery (exploratory laparotomy) or an intraoperative diagnosis of AP or those who had AP subsequent to another disease. All patients signed written informed consent, and this study was performed according to the principles of the Declaration of Helsinki (modified in 2000), and it was approved by the Ethics Committee of Chengdu Military General Hospital (No. 2010017).

### Group division

The patients were divided into two groups based on whether they had undergone APD. The patients in the APD group underwent APD treatment before further necessary interventions were performed, while the patients in the non-APD group did not undergo APD during treatment.

### Management protocols

#### Non-APD group (routine step-up approach)

Both patient groups initially received conservative treatment, such as rigorous fluid resuscitation and gastrointestinal decompression. Nasojejunal enteral feeding and antibiotics were used as necessary. In the non-APD group, when the conservative treatment was not effective and the symptoms deteriorated, the treatment advanced to the second step (percutaneous catheter drainage, PCD) as indicated, similar the procedures described in other reports [14, 15]. The number, size and location of the catheters were determined by the size, viscosity and location of the necrosis, respectively.. The entire procedure was conducted with the cooperation of clinicians and interventional ultrasonographers. If there was no clinical improvement after the initial PCD, additional catheters were placed or replaced. Two professional clinicians and two intervention radiologists assessed the effectiveness of the PCD procedure. If the PCD treatment yielded no clinical improvement, treatment advanced to the third step – open surgery – to eliminate the debris and collections.

#### APD group (modified step-up approach)

A new step, APD, was added for this group after contrast-enhanced computed tomography (CECT) or ultrasound detected abdominal or pelvic fluid collections. The procedure was usually performed within two weeks after the disease onset. APD involved the placement of a tube to persistently drain the collections from the abdominal or pelvic cavity. During the treatment phase, tubes were replaced or added if the initial APD was not competent. PCD was performed as an additional step in a manner similar to other reports [14, 15], and surgery was performed if there was no clinical improvement or if necrosis continued after PCD [15].

#### Procedures and indications of APD (differences between APD and PCD)

APD, a new concept that we created, proved in our previous study to be beneficial to the SAP treatment strategy because of its supplementary effect when administered prior to PCD [1]. Although the surgical steps are similar to those of PCD, there are some differences between the procedures: First, the APD punctures are performed as early as possible, usually within two weeks after the onset of symptoms [1, 2], while PCD is usually performed four weeks after onset to ensure that there is enough necrosis to justify the procedure [13, 14]. Second, the APD puncture sites are in the abdominal or pelvic cavity, usually the right paracolic sulci and left paracolic sulci, while the PCD sites are in the retroperitoneal space (i.e., the peripancreatic region, left pararenal region, or right pararenal region). Finally and most importantly, APD and PCD serve different purposes: APD is performed to remove fluid collections in the abdominal or pelvic cavity and thus relieve the inflammation and organ injury at the early stage of disease, while PCD mainly targets the elimination of peripancreatic necrotic collections in the retroperitoneum to avoid or treat infected necrotizing pancreatitis (INP). Accordingly, the indications of the two interventions are different: whenever there is an appropriately high volume of collections (more than 50-100 ml) and a reachable pathway can be detected through imaging (usually ultrasound), APD can performed. In contrast, PCD is performed in patients who do not demonstrate any improvement or who deteriorate with routine treatment (as indicated by persistent fever, increased leukocyte counts or an increasing trend of leukocyte counts, or worsening or new-onset organ failure, or a diagnosis of INP through CECT or fine-needle aspiration).

#### Indications for surgery

At our center, surgery, such as necrosectomy, was performed after PCD for patients with the following indications: persistent or worsening sepsis after PCD, worsening or new-onset organ failure, inadequate drainage (<10 ml/day) of collections and necrosis, and bowel complications (such as obstruction or uncontrolled fistula) caused by ongoing necrosis [15].

### Data collection

First, we collected and analyzed the differences between the APD group and the non-APD group in terms of baseline data including age, sex, body mass index (BMI) and the levels of lipid metabolites (TG, FFA, cholesterol, lipase) before treatment. Then, we specifically analyzed the changes in lipid metabolites levels in plasma after 2 weeks of treatment. In addition to mortality, days in the hospital and detailed information regarding further interventions were collected as primary clinical outcomes and were analyzed between the two groups. Meanwhile, we analyzed other observational parameters associated with outcomes, such as severity score, infectious factor (white blood cells, procalcitonin, etc.); inflammation factors, including tumor necrosis factor-α (TNF-α), interleukins (ILs), C-reaction protein (CRP); and organ failure-related parameters at 2 weeks after treatment.

All laboratory investigations were conducted within 48 h of admission, at 2 weeks after treatment and occasionally thereafter as indicated. CECT was performed within the first week of the onset of disease, and it was repeated at 2 weeks after treatment. Two experienced radiologists calculated and assessed the CTSI scores obtained from the CECT images. The acute physiology and chronic health evaluation II (APACHE II) score, Ranson score and Marshall score were also serially calculated within 48 h after admission and again at 2 weeks after treatment.

### Measurement of FFA determination

Blood samples were collected and centrifuged, and the serum was stored at -70 °C until analyzed. The total FFA concentration was measured using the enzymatic method (Free Fatty Acid Quantitation Kit, Sigma Chemical Co., St. Louis, MO, USA). The individual FFA measurements were conducted as follows: (1) extraction of lipids from the serum using the Folch and Lees method [16], (2) separation of lipid fractions using thin-layer chromatography, (3) methylation of fatty acids, and (4) separation of fatty acid methyl esters with gas liquid chromatography (CP-3800GC, Varian, Japan) using a 30-m capillary column. In this procedure, helium was used as the carrier gas, and pentadecanoic acid was added as an internal standard [17].

### Statistical analysis

The statistical analysis was performed using SPSS, version 17.0 for Windows (SPSS, Inc., Chicago, IL, USA). The normality of the data was determined using *Kolmogorov-Smirnov* normality tests. The data were expressed as the mean ± standard deviation for normally distributed data and as median and interquartile ranges for non-normally distributed data. For normally distributed data, the variables were compared using *Student’s t* test for two groups. For skewed data, the *Mann-Whitney U* test was used. Qualitative or categorical variables were described as frequencies and proportions. Proportional variables were compared using the *χ2* test or *Fisher’s* exact test. Logistic regression was adopted to assess the factors that were significant for predicting outcomes after 2 weeks of treatment. All statistical tests were two-tailed and were performed at a significance level of *P* < 0.05.

## Results

### General information and baseline data

A total of 272 non-HTG-induced SAP patients with fluid collections were admitted to our center between May 2010 and May 2015; of these, 188 cases (69.12 %) were accompanied with elevated TG. Among the patients with elevated TG, 56 were excluded from the study according to the excluding criteria. Finally, 132 patients were enrolled in the study, of which 64 received routine management or sequential PCD and necrosectomy if necessary (the non-APD group), and 68 received APD or sequential APD and PCD intervention before surgery if necessary (the APD group).

The demographic data of the APD group and the non-APD group were comparable, as Table 1 shows. In both groups, the mean levels of TG and other lipid metabolites (total FFA, cholesterol and lipase) were all abnormally elevated, but there were no significant differences between the two groups. In terms of indicators for body obesity, the body mass index (BMI), waist-to-hip (WHR) and waist circumference (WC) of both groups were higher than normal values, but there were no between-group differences. Meanwhile, the initial severity scores of the APD group were similar to those of the non-APD group. Furthermore, the plasma laboratory parameters (CRP, IL-1β, IL-6, IL-10 and TNF-α, measured 48 h after the onset of the inflammation factors) were all abnormally elevated, and there were no differences between the APD and non-APD groups. Taken together, these results show that in addition to the abnormal elevation of inflammation factors, there was also an abnormal elevation of FFA in the non-HTG-induced SAP patients with TG elevation.

**Table 1:** The characteristics of 132 patients enrolled in this study

Characteristic	APD group	Non-APD group	*t* or *χ* ^2^	*P* value
	*n* = 68	*n* = 64	value	
Demographic data (mean ± SD)				
Age	50 .33 ± 11.17	48.79 ± 9.54	0.849	0.397
Gender			0.129	0.72
Male	34	34		
Female	34	30		
BMI(kg/m^2^)	26.72 ± 1.96	27.19 ± 1.65	1.675	0.096
waist-to-hip (WHR)	1.18 ± 0.08	1.19 ± 0.11	0.600	0.550
waist circumference (WC)(cm)	107.1 ± 12.9	113.5 ± 18.6	0.540	0.479
Lipid metabolites levels of serum				
TG (mmol/L)	6.83 ± 2.03	7.12 ± 1.57	0.914	0.362
Total FFA (mmol/L)	1.77 ± 0.22	1.80 ± 0.31	0.644	0.521
Cholesterin (mmol/L)	5.20 ± 0.81	5.07 ± 0.87	0.889	0.376
Lipase (U/L)	1220 ± 277	1195 ± 301	0.497	0.620
Initial severity scores (mean ± SD)				
APACHE II score	15.21 ± 3.48(8–65)	15.47 ± 3.10(8–62)	0.452	0.652
CTSI score	8.15 ± 2.87(6–10)	8.22 ± 3.06(6–12)	0.136	0.892
Ranson score	3.27 ± 1.47(1–8)	3.10 ± 1.35(1–8)	0.691	0.491
Marshall score	4.15 ± 1.74(2–6)	4.19 ± 1.23(2–6)	0.152	0.880
Laboratory parameters (mean ± SD)				
CRP (mg/L)	143.29 ± 19.73	142.81 ± 20.91	0.136	0.892
IL-1β (pg/L)	14.70 ± 1.21	15.17 ± 2.17	1.232	0.220
IL-6 (pg/L)	351.78 ± 63.21	354.27 ± 50.83	0.248	0.804
IL-10 (pg/L)	149.11 ± 51.39	143.21 ± 50.09	0.667	0.506
TNF-α (pg/L)	19.18 ± 5.33	18.35 ± 4.99	0.922	0.358

*Abbreviations*: *APD* abdominal paracentesis drainage, *PCD* percutaneous catheter drainage

*BMI* body mass index, *WHR* waist-to-hip ratio, *WC* waist circumference, *CRP* C-reaction protein, *IL* interleukin, *TNF-α* tumor necrosis factor-alpha, *TG* triglycerides, *FFA* free fatty acids, *APACHE II* Acute Physiology and Chronic Health Evaluation II, *CTSI* computerized tomography severity index, *APD group* patients in this group treated with APD, *Non-APD group* patients in this group treated without APD

### Primary clinical outcomes in the APD and non-APD groups

Detail information about the primary clinical outcomes after treatment for the APD and non-APD groups is shown in Table 2. The mortality rate in the APD-group was significantly lower than that of the non-APD group (5/68, 7.35 % vs 9/64, 14.06 %, *P* < 0.05). The length of hospital stay in the APD group was shorter than that of the non-APD group (52.78 ± 22.51 vs 66.13 ± 33.07, *P* < 0.05). Before the PCD operation became necessary and was performed, 23 patients in the APD group (23/68, 33.82 %) demonstrated clinical improvement, which was significantly more than those who received conservative treatment only in the non-APD group (12/64, 18.75 %). In terms of the PCD details, first, the PCD schedule was significantly altered in the APD group compared with the non-APD group, and the interval between the onset of disease and the first PCD insertion was significantly longer in the APD group compared with the non-APD group (34.18 ± 5.23 vs 26.73 ± 6.05, *P* < 0.001). Second, although the differences were not significant, the PCD duration was shorter (35.41 ± 7.57 vs 39.37 ± 12.05, *P* = 0.060) and the total number of PCD procedures per patient was smaller (4.57 ± 2.35 vs 5.40 ± 2.19, *P* = 0.075) in the APD group compared with the non-APD group. These results suggest that, in non-HTG-induced SAP patients with elevated serum TG, the APD procedure could postpone or even avoid further intervention, thus minimizing the ‘second hit’ that accompanies intervention and consequently reducing both the mortality rate and the length of hospital stay. Taken together, these results indicate that treatment with APD benefited the primary clinical outcomes of the non-HTG-induced SAP patients with TG elevation.

**Table 2:** The primary clinical outcomes in the APD and non-APD groups

	APD group	Non-APD group	*t* or *χ* ^2^	*P* value
	*n* = 68	*n* = 64	value	
Mortality rate	5/68 (7.35 %)	9/64 (14.06 %)	3.897	0.042^*^
Hospital stay, days	52.78 ± 22.51	66.13 ± 33.07	4.587	0.037^*^
Need for further intervention			3.901	0.048^*^
Negative	23	12		
Positive	45	52		
PCD				
Interval between the onset of symptoms and the first PCD insertion, days	34.18 ± 5.23	26.73 ± 6.05	6.437	<0.001^*^
Duration of PCD, days	35.41 ± 7.57	39.37 ± 12.05	1.903	0.060
Total NO. Of PCD procedures per patient	4.57 ± 2.35	5.40 ± 2.19	1.799	0.075

*Abbreviations*: *APD* abdominal paracentesis drainage, *PCD* percutaneous catheter drainage

APD-group = patients in this group treated with APD; non-APD group = patients in this group treated without APD. ^*^Significant difference

### Organ failure, infection-related parameters, and inflammatory factors in the APD and non-APD groups after treatment

The parameters related to organ failure, infections and inflammation were collected and analyzed after 2 weeks of treatment (Table 3). Compared with the non-APD group, in the APD group the duration of organ failure was shorter (21.29 ± 4.57 vs 23.34 ± 5.33, *P* = 0.049) and the recurrence rate of organ failure was lower (11, 30.5 % vs 16, 43.2 %, *P* < 0.05). Moreover, in terms of the supporting treatment details, there were fewer instances of mechanical ventilation (29 vs 59, *P* < 0.001) in the APD group compared with the non-APD group, and the mean duration of supporting treatment was significantly shorter in the APD group (5.27 ± 2.77 vs 7.91 ± 3.05, *P* < 0.001). These results show that the APD group had a lower rate of organ failure recurrence and required less time for the reversal of organ failure.

**Table 3:** Organ failure- and infection-related parameters and inflammatory factors of the APD and non-APD groups

	APD group	Non-APD group	*t* or *χ* ^2^	*P* value
	*n* = 68	*n* = 64	value	
Organ failure				
Duration of organ failure, days	21.29 ± 4.57	23.34 ± 5.33	1.993	0.049^*^
Recurrence rate of organ failure	11 (30.5 %)	16 (43.2 %)	3.410	<0.05^*^
Mechanical ventilation, *N*	29	59	36.412	<0.001^*^
Duration of mechanical ventilation treatment	5.27 ± 2.77	7.91 ± 3.05	3.930	<0.001^*^
Infection				
Incidence of infections	47	46	0.201	0.973
White blood cell count (×10^9^/L)	13.27 ± 3.19	13.25 ± 3.01	0.754	0.518
Procalcitonin > 5 ng/ml	44/68, 64.7 %	39/64, 60.9 %	0.873	0.134
Reversal time of sepsis, days	18.14 ± 2.83	23.76 ± 4.34	2.690	<0.05^*^
Inflammatory factors				
CRP (mg/L)	76.5 ± 22.73	103.5 ± 31.09	34.274	<0.001^*^
IL-1β (pg/L)	5.33 ± 1.35	9.97 ± 3.84	13.401	<0.001^*^
IL-10 (pg/L)	58.7 ± 16.32	65.3 ± 14.16	1.782	0.077
TNF-α (pg/L)	10.1 ± 1.07	15.2 ± 2.19	10.954	<0.001^*^

*Abbreviations*: *APD* abdominal paracentesis drainage, *PCD* percutaneous catheter drainage, *CRP* C-reaction protein, *IL* interleukin, T*NF-α* tumor necrosis factor-alpha, *APD group* patients in this group treated with APD, *non-APD group* patients in this group treated without APD. *Significant difference

We estimated the occurrence of infection based on the incidence of infection, levels of infectious laboratory parameters and the time required to reverse sepsis. The incidence of infection in the APD group had no significant change compared with that of the non-APD group (47, 69.11 % vs 46, 71.88 %, *P* > 0.05). The white blood cell counts and procalcitonin levels did not vary greatly between the two groups after treatment. Moreover, for patients with sepsis, the time required to reverse was shorter (18.14 ± 2.83 vs 23.76 ± 4.34, *P* < 0.05) in the APD group compared with the non-APD group.

Inflammatory factors, such as CRP, IL-1β, IL-10 and TNF-α were measured to analyze the inflammatory state. In contrast with the non-APD group, the APD group had significantly lower levels of CRP (76.5 ± 22.73 vs 103.5 ± 31.09, *P* < 0.001), IL-1β (5.33 ± 1.35 vs 9.97 ± 3.84, *P* < 0.001), IL-10 (58.7 ± 16.32 vs 65.3 ± 14.16, *P* = 0.077) and TNF-α (10.1 ± 1.07 vs 15.2 ± 2.19, *P* < 0.001). These results indicated that the APD group had a more improved inflammatory state compared with the non-APD group after treatment.

### The variation in severity scores after treatment

**Fig. 1:**
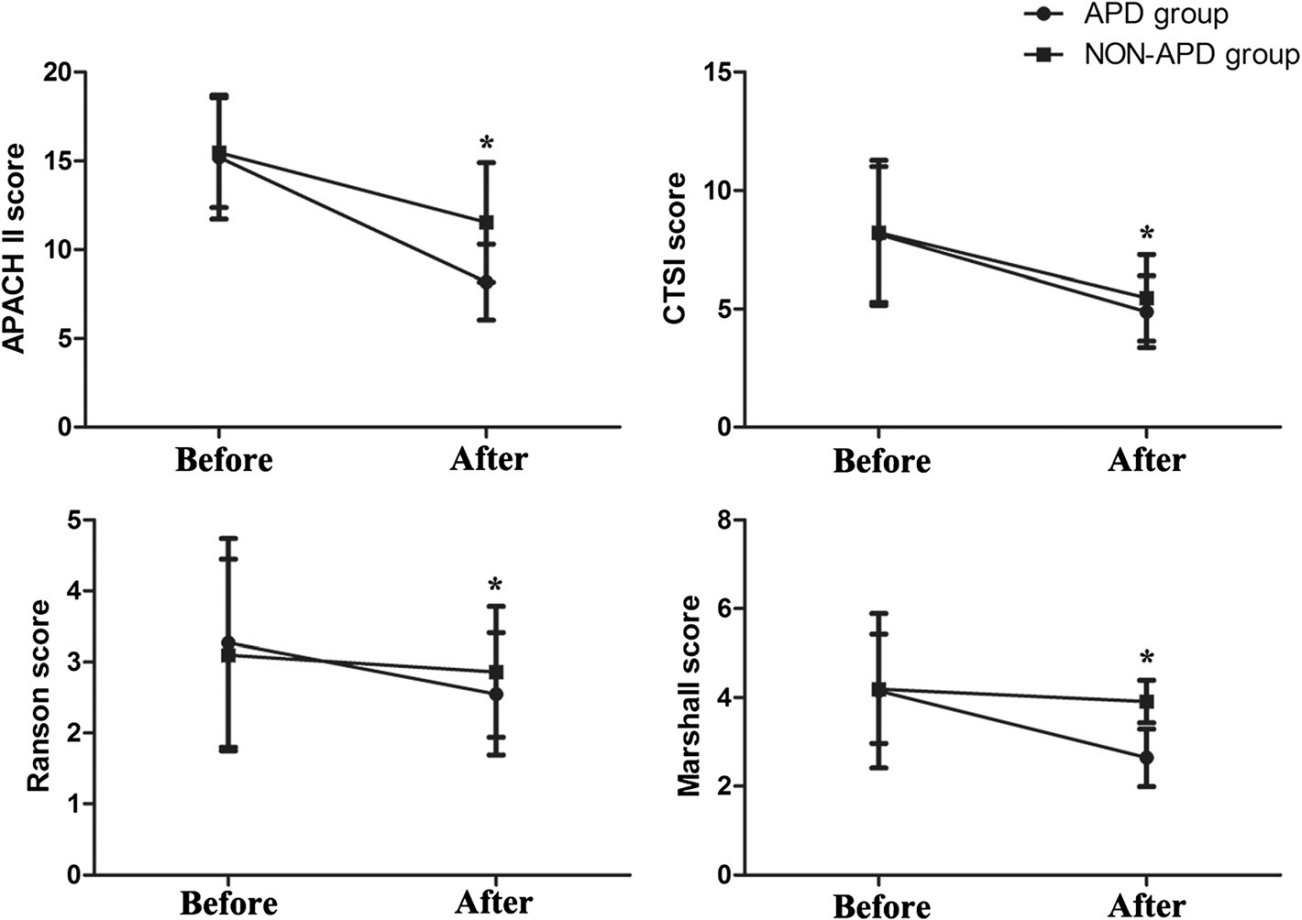
Variation of the severity scores before and after treatment. The severity scores were decreased in both groups after two types of conservative treatment; moreover, the severity scores of the APD group were lower than that those of the non-APD group

We employed the APACHE II, Ranson, Marshall and CTSI scoring systems to determine the severity of disease at 2 weeks after treatment. All scores in both groups were decreased after the two types of treatment. However, the APACHE II scores (8.17 ± 2.13 vs 11.54 ± 3.37, *P* < 0.001), Ranson scores (2.55 ± 0.86 vs 2.86 ± 0.92, *P* = 0.047), Marshall scores (2.64 ± 0.65 vs 3.91 ± 0.48, *P* < 0.001) and CTSI scores (4.88 ± 1.51 vs 5.47 ± 1.83, *P* = 0.045) were lower in the APD group compared with the non-APD group (Fig. 1).

These results indicate that APD could relieve the severity state and reverse the deterioration of progress in the early stage of SAP, similar to our previous findings.

### The change in lipid metabolites after treatment

**Fig. 2:**
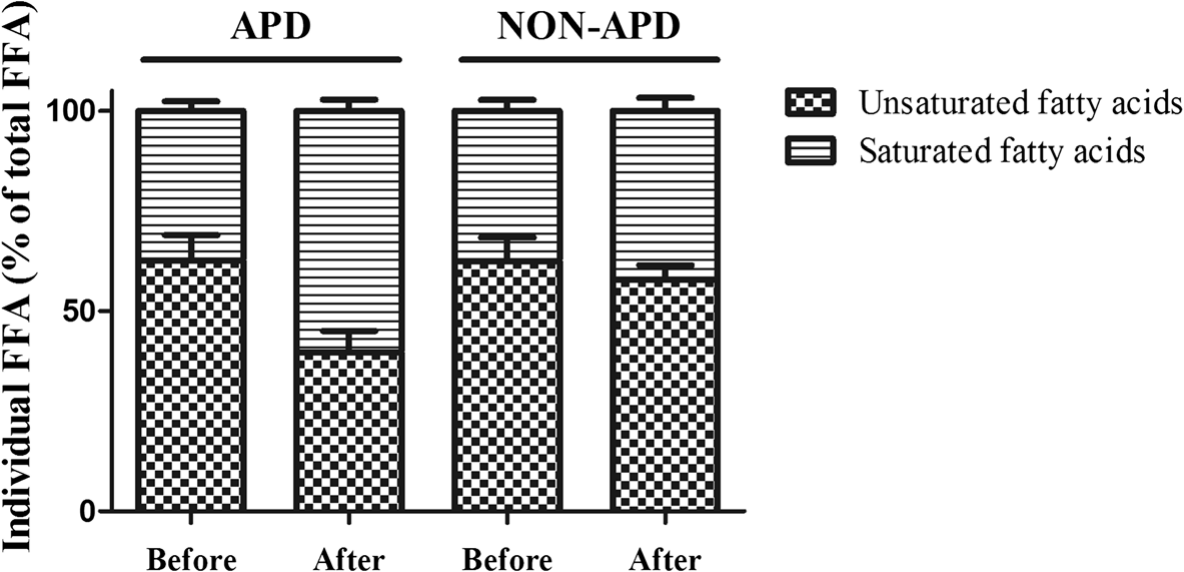
The variation in FFA profiles before and after treatment in both groups. The percentage of UFA decreased in both groups, and the extent of the decrease was greater in the APD group than in the non-APD group (62.67 % vs 39.66 %, *P* < 0.001)

The concentrations of lipid metabolites, mainly TG and FFA, decreased after 2 weeks of treatment in both groups (Table 4). However, the serum levels of TG (2.45 ± 1.07 vs 5.08±2.21, *P* < 0.001) and total FFA (0.67±0.15 vs 1.33±0.12, *P* < 0.001) in the APD group were significantly lower than those in the non-APD group. Cholesterol and lipase had slightly lower expression levels in the APD group compared with the non-APD group, although these differences were not significant. Furthermore, compared with the non-APD group, the patients in the APD group showed more obvious variation in their FFA profiles (Table 4 and Fig. 1). In terms of the proportional contribution of individual FFA to the total FFA, compared with the initial FFA profiles, the percentage of unsaturated fatty acids (UFA) showed a downward trend with increased proportions of saturated fatty acids (SFA) in both groups after treatment (Fig. 2). Remarkably, the proportional contribution of UFA in the APD group decreased significantly compared with that of the non-APD group (62.67 % vs 39.66 %, *P* < 0.05).

**Table 4:** The serum levels of metabolites in the APD and non-APD groups after treatments

	APD group	Non-APD group	*t* value	*P* value
	*n* = 68	*n* = 64		
Lipid metabolites				
TG (mmol/L)	2.45 ± 1.07	5.08 ± 2.21	15.401	<0.001^*^
Total FFA (mmol/L)	0.67 ± 0.15	1.33 ± 0.12	10.954	<0.001^*^
Cholesterin (mmol/L)	2.88 ± 0.74	3.01 ± 0.68	1.049	0.296
Lipase (U/L)	195 ± 47	211 ± 56	1.782	0.077
FFA profiles (%)				
Lauric acid 12:0	0.92 ± 0.33	0.71 ± 0.23	1.655	0.100
Myristic acid 14:0	6.11 ± 1.81	3.05 ± 0.62	7.598	<0.001^*^
Palmitic acid 16:0	32.19 ± 9.63	22.61 ± 5.81	9.693	<0.001^*^
Palmitoleic acid 16:1	4.71 ± 1.57	4.27 ± 2.07	0.157	0.876
Stearic acid 18:0	18.03 ± 5.31	12.50 ± 2.46	10.467	<0.001^*^
Oleic acid 18:1	25.87 ± 4.73	34.52 ± 6.17	12.363	<0.001^*^
Linoleic acid 18:2	8.08 ± 0.52	13.31 ± 1.73	23.817	<0.001^*^
Linolenic acid 18:3	0.33 ± 0.12	0.37 ± 0.18	1.510	0.133
Arachidonic acid 20:4	1.21 ± 0.25	5.41 ± 1.09	6.041	<0.001^*^

*Abbreviations*: *TG* triglycerides, *FFA* free fatty acids, *APD* abdominal paracentesis drainage, *PCD* percutaneous catheter drainage, *APD group* patients in this group treated with APD, *non-APD group* patients in this group treated without APD. *Significant difference

**Fig. 3:**
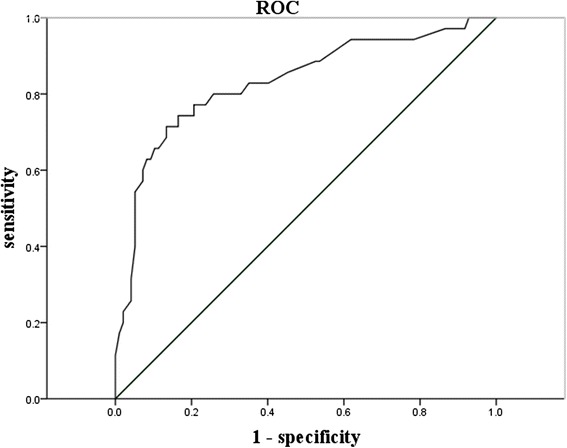
Receiver operating characteristic (ROC) curve. The Δ-Total FFA presented a discrimination with area under the receiver operating characteristic curve of 0.831 (*P* < 0.001, 95 % CI, 0.745–0.917)

To further investigate the correlation between APD and the variation in lipid metabolites levels in plasma, we analyzed the lipid levels of the PAAF collected during the initial APD intervention in the APD group. Compared with normal serum concentrations, high levels of TG and total FFA (TG: 5.17 ± 1.88, FFA: 1.23 ± 0.47) were observed in the drained PAAF. The concentrations of lipid metabolites were not as high as those in plasma; however, this may be due to the consequent dilution of fluids into the abdominal cavity. Taken together, these results suggest that the APD procedure could significantly decrease the levels of TG and FFA in plasma, and these variations in plasma might have a correlation with the drainage of PAAF.

### The decreased extents of TG and total FFA were protective factors

Next, we used univariable and multivariable logistic regression analyses to identify predictors of clinical improvement at 2 weeks after treatment. According to the PANTER trial, "Clinical improvement" is defined as (1) improved function of organ failure or no new-onset organ failure, (2) improvement of two out of the following three parameters: leucocytes/temperature/CRP.

Table 5 shows the factors indicative of clinical improvement after 2 weeks of treatment. The decreased extents (Δ) of TG, Δ-FFA and Δ-TNF-a were significantly different between patients with and without clinical improvement. These factors were described as potential predictors of improvement after treatment and were analyzed as variables in the multivariable logistic regression model. In the multivariable logistic regression analysis, △-FFA was significantly different between patients with and without clinical improvement and thus was identified as protective factor (Table 6).

**Table 5:** Univariable logistic regression analysis of clinical improvement after treatment

Variable		95 % CI OR		
	OR	Lower	Upper	*P* value
Δ-TG	1.672	1.496	2.842	0.017*
Δ-Total FFA	1.650	1.524	1.737	0.034*
Δ-CRP	2.152	2.353	3.891	0.109
Δ-IL-1β	1.796	0.660	1.960	0.017
Δ-TNF-α	1.939	1.887	2.994	0.031*

*Abbreviations*: *TG* triglycerides, *FFA* free fatty acids, *CRP* C-reaction protein, *IL* interleukin, *TNF-α* tumor necrosis factor-alpha, Δ the extent of decreased level after 2 weeks of treatment. *Significant difference

**Table 6:** Multivariable logistic regression analysis of clinical improvement after treatment

		95 % CI OR		
Variable	OR	Lower	Upper	*P* value
Δ-TG	3.672	2.196	6.142	0.070
Δ-Total FFA	1.435	1.124	3.234	0.015*
Δ-TNF-α	1.939	0.887	2.994	0.131

*Abbreviations*: *TG* triglycerides, *FFA* free fatty acids, *TNF-α* tumor necrosis factor-alpha

Δ = the extent of decreased level after 2 weeks of treatment. *Significant difference

For further assessment of the protective effect, the area under the receiver operating characteristic curve (ROC) of Δ-FFA was calculated (Fig. 3). It was found that a Δ-FFA of more than 0.835 mmol/L after 2 weeks of treatment could predict clinical improvement with a sensitivity of 71.0 % and a specificity of 86.6 %. Notably, the value of Δ-FFA in the APD group was 1.07 ± 0.21 mmol/L, which was higher than 0.835 mmol/L. In contrast, the value of Δ-FFA in the non-APD group was only 0.48 ± 0.10 mmol/L. These data indicated the clinical improvement of APD on SAP patients correlated to the decreased extents of total FFA.

## Discussion

In this study, a novel mechanism was proposed that indicates the APD exerts beneficial effects on SAP patients by reducing the serum levels of TG and FFA. The important findings are that (i) APD is beneficial for non-HTG-induced SAP patients with TG elevation; (ii) APD could reduce the level of serum TG and FFA; and (iii) The effectiveness of APD has a clear correlation with the reduction of FFA in plasma. These findings provide new insight into the indications for APD and the mechanisms for its effectiveness, which may advance the clinical efficacy of APD for SAP.

### APD could benefit SAP patients with TG elevation

In recent years, many studies have shown that SAP patients with elevated plasma levels of TG or FFA usually exhibit a trend toward aggravation and have a worse outcome compared with those with normal TG or FFA levels [6, 10–12, 18]. Therefore, effective strategies for treating this subset of patients need to be developed. In our current study, we first determined the clinical efficacy of APD in non-HTG-induced SAP patients with TG elevation and PAAF. Compared with the non-APD group, the APD group showed lower mortality and a shorter mean duration of hospital stay, which suggests that APD could improve the primary outcomes of this subset of SAP patients. Moreover, the APD group showed a lower mean duration of organ failure and supporting treatment and a lower rate of organ failure recurrence compared with the non-APD group, which suggests that APD has a reversal effect on organ failure. The serum levels of inflammatory factors were also significantly decreased in the APD group compared with the non-APD group, which indicates that APD could relieve systemic inflammation. In addition, the APD group showed more improved severity scores after treatment compared with the non-APD group. Furthermore, the need for additional intervention (PCD) was postponed, and the incidence of PCD was lower in the APD group. These results indicate that APD could effectively treat SAP accompanied by serum TG and FFA elevation by ameliorating its clinical outcomes while avoiding additional infection.

### APD could reduce the serum levels of TG and FFA

In our previous report, we found that APD exerts a beneficial effect on SAP patients by reducing intra-abdominal pressure (IAP) [3]. Considering that not all SAP patients present high IAP [19–21] and that multiple causes are involved in the development of SAP [22], it is necessary to determine whether and which other factors could be relieved or eliminated during the APD procedure. Numerous studies have revealed the types of substances that are elevated in the PAAF of SAP patients and in the animal model of AP [23], including adipokines [24]; proteases [25, 26]; cytokines, such as interleukin (IL)-1β [27], IL-6 [28] and IL-8 [29]; and lipid metabolites, such as TG and FFA [5, 7, 30, 31]. In our present study, we demonstrated that APD could significantly decrease the serum levels of cytokines, including CRP, IL-1β, IL-6 and TNF-α, which is consistent with previous studies [1]. Notably, we found a drastic decrease of lipid metabolites such as TG and FFA after APD treatment. This is supported by some published evidence that lipid metabolites such as TG and FFA, which are enriched in PAAF, seem to play a role in the pathogenesis of SAP [5, 6, 32]. For example, in a clinical study, Vijay and his colleagues found an enrichment of FFA in PAAF. Meanwhile, they found that increased lipid metabolites, especially UFA, but not IL-1β or IL-8, could cause multi-system organ failure by inducing necro-apoptosis in animal models of AP [6, 32]. In addition, Closa and his colleagues revealed that FFA extracted from PAAF could affect liver function after gaining access to portal system and exacerbate systemic inflammation in pancreatitis by interfering with the endogenous regulatory mechanism of the inflammatory factors upon the activation of NF-kB in local macrophages. Taken together, our data demonstrate that APD treatment could drastically decrease the serum levels of TG and FFA, which may play the important role in improving the clinical outcomes of SAP patients with TG elevation [5].

### The decreased extent of FFA is correlated with the clinical improvement of SAP

To date, the relationship between TG/FFA and the pathogenesis of pancreatitis requires further documentation. In the present study, univariable and multivariable logistic regression analyses showed that the decreased extent of FFA in plasma after treatment was an independent protective factor for the clinical improvement of SAP. Furthermore, the ROC curve indicated that a △-FFA of more than 0.835 mmol/L correlated with the outcomes after treatment. In particular, treatment with APD leads to greater decreases in serum FFA (APD group: 1.07 ± 0.21 mmol/L vs non-APD group 0.48 ± 0.10 mmol/L, P < 0.05), thereby benefiting the clinical improvement of SAP.

### How does APD reduce the levels of TG and FFA in plasma?

The most important finding of this study is that both TG and FFA could be significantly reduced by APD. One may ask how APD reduces the levels of TG and FFA in plasma. A possible explanation is that APD could decrease the serum levels of TG and FFA via the drainage of PAAF. In addition, through the analysis of the FFA profiles, we found that the serum levels of UFA, which could be generated by fat necrosis [6, 32], decreased significantly in the APD group compared with the non-APD group. Based on these results, we propose APD could reduce the TG and FFA levels in two ways: by eliminating PAAF directly or by ameliorating the fat degeneration and thus reducing the reactive products indirectly.

### Why does abnormal elevation of FFA emerge in non-HTG-induced SAP patients?

Another important observation of this study is that the concentration of total FFA in plasma was greatly elevated and was accompanied by the elevation of TG in non-HTG-induced SAP patients. Possible explanations for this result are that (i) the excess amounts of TG in the circulation are hydrolyzed by high levels of pancreatic lipase, thus resulting in the very high concentration of FFA [8] and (ii) a large amount of FFA is generated via fat necrosis [32]. However, under any conditions, the concentration of FFA might inevitably exceed the binding capacity of plasma albumin and finally self-aggregate to FFA micelles, which are responsible for pancreatic ischemia and vessel necrosis [33].

## Conclusion

In conclusion, we found that APD is beneficial for non-HTG-induced SAP patients with elevated TG and PAAF. Notably, we revealed a novel mechanism through which the reduction of serum TG and FFA may account for the beneficial effect. Our findings provide new insight into the indications for APD and mechanisms of its effectiveness, which may advance the clinical efficacy of APD for SAP.

### Limitations and future study

Because of its retrospective design, this study has some limitations. First, it is difficult to avoid a data bias from the analysis because of derandomization. Second, the serum levels of TG and FFA vary in cases of pancreatitis with different etiologies and in different phases of treatment. When selecting cases, we excluded patients with a history of hyperlipidemia or alcoholism in an effort to exclude factors other than fat necrosis that might generate TG and FFA. Moreover, patients who had undergone routine antihyperlipidemic therapy throughout treatment were also excluded. Nonetheless, nutritional support during the treatment might have partly influenced the results. In addition, though we recommended APD as an optimal intervention for SAP patients with PAAF in the early stage of disease in current and previous studies, there is a need for prospective research to further determine the indications for APD.

## Abbreviations

APACHE II, Acute physiology and chronic health evaluation II; APD, Abdominal Paracentesis Drainage; BMI, body mass index; CRP, C-reaction protein; CTSI, computed tomography severity index; FFA, free fatty acid; HTG, hypertriglyceridemia; IAH, intra-abdominal hypertensionIL, interleukin; INP, infected necrotizing pancreatitis; PAAF, pancreatitis associated ascitic fluid; PCD, percutaneous catheter drainage; ROC, receiver operating characteristic curve; SAP, severe acute pancreatitis; TG, triglycerides; TNF-α, tumor necrosis factor-alpha; UFA, unsaturated fatty acids; WC, Waist circumference; WHR, waist-to-hip
